# Detection of *sasX* Gene and Distribution of SCC*mec* Types in Invasive and Non-invasive Coagulase-negative Staphylococci

**DOI:** 10.4274/balkanmedj.galenos.2020.2019.8.21

**Published:** 2020-06-01

**Authors:** Alper Tekeli, Duygu Nilüfer Öcal, İştar Dolapçı

**Affiliations:** 1 Department of Medical Microbiology, Ankara University School of Medicine, Ankara, Turkey

**Keywords:** Antimicrobial resistance, coagulase-negative staphylococci, molecular epidemiology, sasX, SCCmec

## Abstract

**Background:**

Coagulase-negative staphylococci, which belong to the normal microbiota of the skin and mucous membranes, are opportunistic pathogens. *sasX*, a newly described protein, is thought to play an important role in nasal colonization and methicillin-resistant *Staphylococcus aureus* virulence, and it may be acquired from coagulase-negative staphylococci by horizontal gene transfer. It has been considered that understanding the function of *sasX* gene may help clarify the relevance of the different adhesion mechanisms in the pathogenesis of infections associated with biofilm.

**Aims:**

To investigate the *sasX* gene presence, staphylococcal cassette chromosome *mec* types, and antimicrobial resistance patterns of invasive and noninvasive coagulase-negative staphylococci isolates.

**Study Design:**

Cross-sectional study.

**Methods:**

The study included a total of 180 coagulase-negative staphylococci strains. Non-invasive isolates (n=91) were obtained from the hands of healthy volunteers who do not work at the hospital (n=30), the nasal vestibule of healthy volunteer hospital workers (n=26), and central venous catheter (n=35). Invasive isolates (n=89) were isolated from peripheral blood cultures of inpatients who do not have catheters. All isolates were identified by conventional microbiological methods, automated systems, and, if needed, with matrix-assisted laser desorption/ionization-time of flight. Staphylococcal cassette chromosome *mec* typing, *sasX* and *mec* gene detection, antibiotic susceptibility, and *sasX* gene sequence analysis were performed.

**Results:**

Peripheral blood, central venous catheter colonization, and nasal vestibule isolates were positive for the *sasX* gene, whereas hand isolates were negative. *sasX* gene was present in 17 isolates, and no statistical significance was found between invasive and noninvasive isolates (p=0.173). Sequence analysis of the *sasX* genes showed high homology to related proteins of *Staphylococcus* phage SPbeta-like and *Staphylococcus epidermidis* RP62A. staphylococcal cassette chromosome *mec* type V was the most prevalent regardless of species. staphylococcal cassette chromosome *mec* type II was more frequent in invasive isolates and found to be statistically important for invasive and noninvasive *S. epidermidis* isolates (p=0.029). *Staphylococcus haemolyticus* isolates had the overall highest resistance rates. Resistance to ciprofloxacin, trimethoprim-sulfamethoxazole, and erythromycin was found to be higher in isolates from catheter and blood culture. *Staphylococcus hominis* isolates had the highest rate for inducible clindamycin resistance. None of the isolates were resistant to vancomycin, teicoplanin, and linezolid.

**Conclusion:**

The sasX gene is detected in 9.44% of the isolates. There is no statistical difference between the *sasX*-positive and -negative isolates in terms of antibacterial resistance and the presence of *sasX* and SCC*mec* types. Further studies about the role of *sasX* at virulence in coagulase-negative staphylococci, especially from clinical samples such as tracheal aspirate and abscess isolates, and distribution of staphylococcal cassette chromosome *mec* types are needed.

Coagulase-negative staphylococci (CoNS), which belong to the normal microbiota of the skin and mucous membranes, are opportunistic pathogens, particularly in patients who are immunocompromised or have indwelling devices (1). Especially, *Staphylococcus epidermidis* and *Staphylococcus haemolyticus* are considered to be the major nosocomial pathogens, as well as others like *Staphylococcus saprophyticus* (2,3). These bacteria are also the cause of bacteremia in nearly 30% of bloodstream-related infections and responsible for sepsis episodes in immunocompromised patients (4).

Resistance to methicillin and almost all beta-lactam antibiotics in staphylococci is mostly caused by the acquisition of the *mecA *gene, encoding an additional altered low-affinity penicillin-binding protein, which is located on a mobile genetic island, termed the staphylococcal cassette chromosome *mec* (SCC*mec*). SCC*mec* contains *mec* gene complex and *ccr* gene complex of varying size and genetic structure (5). There is strong evidence suggesting the diversity of SCC*mec* in CoNS. New combinations of a *mec* complex and *ccr* complex, differences from the amplification of the *ccr* gene, and multiple presence of *ccr* gene complex make the typing difficult in CoNS (6,7,8,9).

*sasX*, a homolog of *sesI*, is a newly described protein that is thought to play an important role in nasal colonization and methicillin-resistant *Staphylococcus aureus* (MRSA) virulence, and *sasX* gene is located on a ϕSPβ-like prophage (10). *sesI* is one of the *S. epidermidis* surface (ses) proteins that can be expressed during biofilm expression (11) and is encoded by ϕSPβ prophage (12). The sequence similarity of *sesI* and *sasX* genes indicates that the latter may be acquired from CoNS by horizontal gene transfer, and it has been considered that understanding the function of *sasX* gene may help clarify the relevance of the different adhesion mechanisms in the pathogenesis of infections associated with biofilm (13).

This study aimed to investigate the *sasX* gene carriage, which was thought to play a role at attachment, pathogenicity, and distribution of the SCC*mec* types of invasive and noninvasive CoNS.

## MATERIALS AND METHODS

### Bacterial strains and identification

A total of 180 CoNS strains from İbni Sina Hospital Central Microbiology Laboratory Biobank, which were previously approved by the ethical committee for clinical investigations of Ankara University School of Medicine - 21-680-12), were included in the study. Noninvasive isolates (n=91) were obtained between 2013 and 2014 from the hands of healthy volunteers who do not work at the hospital without chronic diseases (n=30), noses of healthy volunteers (nurses, doctors, and hospital staff) who worked at the university hospital (902 beads) without chronic diseases (n*=*26), and central venous catheter colonization (n*=*35). Catheter colonization is defined as the growth of an organism, using the semiquantitative roll-plate culture technique greater than 15 colony-forming units from the tip or the subcutaneous segment of the removed catheter in patients who have no growth at their blood cultures, which were taken simultaneously (14). On the other hand, invasive isolates (n=89) were isolated from peripheral blood cultures of inpatients who do not have catheters. In our study, we defined isolates from sterile body sites, including blood (which were causative agents of bloodstream infections according to guidelines) as invasive and isolates from hands, nasal vestibules, and central venous catheter colonization as noninvasive. Isolates were identified by conventional microbiological methods (Gram staining, catalase, and coagulase tests), BD Phoenix (Becton Dickinson, USA) automated systems, and, if needed, Bruker Microflex MS (Bruker Daltonics, Bremen, Germany).

### Susceptibility testing

A disk diffusion test determined the susceptibilities of isolate to cefazolin 30 μg, amoxicillin-clavulanate 20/10 μg, rifampicin 5 μg, cefoxitin 30 μg, tetracycline 30 μg, clindamycin 2 μg, erythromycin 15 μg, trimethoprim-sulfamethoxazole 1.25/23.75 μg, gentamicin 10 μg, linezolid 30 μg, and teicoplanin 30 μg, and broth microdilution method for vancomycin minimum inhibitor concentrations (MIC). The results were evaluated according to the Clinical and Laboratory Standards Institute standards (15). Isolates that were resistant to cefoxitin were regarded as methicillin resistant.

### *sasX, mecA* detection, and SCC*mec* Typing

Standard polymerase chain reaction (PCR) detected *sasX* and *mecA* genes (10,16) and multiplex PCR strategy for SCC*mec* types as previously described Kondo et al. (17).

### Sequence analysis of *sasX* gene

Sequencing of *sasX* genes was performed using ABI Prism 310 genetic analyzer (Applied Biosystems, Foster City, CA, USA) with BigDye fluorescent terminator chemistry. Sequence data obtained were analyzed with the basic local alignment search tool of the National Center for Biotechnology Information (18).

### Statistical analysis

The Kruskal-Wallis variance analysis compared the vancomycin MIC values, and chi-square or Fisher’s exact tests for all other comparisons, as appropriate. All analyses were performed using SPSS Statistics for Windows, Version 15.0. A p-value <0.05 was considered statistically significant.

## RESULTS

### Bacterial strains

Of the 180 isolates, *S. epidermis* (n=60), *S. haemolyticus* (n=48), *S. hominis *(n=46), *Staphylococcus capitis* (n=13), *Staphylococcus warneri* (n=8), *Staphylococcus lugdunensis* (n=2), *S. saprophyticus* (n=2), and *Staphylococcus scuiri* (n=1) were isolated. Distribution of the strains according to the sample types are given in Table 1.

### Antimicrobial susceptibility patterns

Methicillin resistance was found in 135 of the isolates using the cefoxitin disk diffusion test. When overall antibiotic resistance was considered, *S. haemolyticus* isolates had the highest resistance rates except for clindamycin and tetracycline (Table 2). Resistance to ciprofloxacin, trimethoprim-sulfamethoxazole, and erythromycin was found to be higher in isolates from catheter colonization (71.4%, 71.4%, and 80%, respectively) and blood culture (70.8%, 68.5%, and 85.5%, respectively). Considerable proportions of isolates from the nose were found to be susceptible to ciprofloxacin (96.2%), trimethoprim-sulfamethoxazole (96.2%), and rifampicin (96.2 %). *S. hominis* isolates had the highest inducible clindamycin resistance rates (36.9%), followed by *S. capitis* (23.07%), *S. epidermidis* (11.7%), and *S. haemolyticus* (8.3%). None of the isolates were found to be resistant to vancomycin, teicoplanin, and linezolid. The resistance patterns of the isolates are given in Table 2. Vancomycin MIC50, MIC90, and MIC ranges were 1, 4, and 0.125-4 μg/mL, respectively, by broth microdilution method, and the minimum, median, and maximum values of vancomycin for the CoNS spp. are given in Table 3. MIC ranges of the samples are given in Table 4. No difference was found between vancomycin MIC values and the isolates (p=0.477) and SCC*mec* types (p=0.277). No difference was found between *sasX*-positive and negative isolates in terms of antibiotic resistance patterns.

### Presence of *sasX* and *mecA* genes

*sasX* gene was detected in 9.44% (17/180) of the isolates. Of the strains isolated, 12.36% (n=89) from peripheral blood, 11.42% (n=35) from catheter colonization, and 7.69% (n=26) from nasal vestibules were positive for the *sasX* gene, whereas strains from healthy hands (n=30) were negative. *sasX* carriage rate distribution and p-values according to the sample types are presented in Table 5.

Of the 17 *sasX*-positive strains (17/180), nine were *S. haemolyticus *(9/48, 18.42%), four *S. epidermidis *(4/60, 6.66%), three *S. hominis *(3/46, 6.52%), and one *S. capitis *(1/13, 7.69%). *S. warneri*,* S. lugdunensis*, *S. saprophyticus*, and *S. sciuri* isolates were found to be negative for the *sasX *gene, and no statistical significance was found between invasive and noninvasive isolates (p=0.173).

Of the 180 isolates,* mecA *carriage was found as 75% (n=135). *mecA ratios *of the strains were as follows: *S. haemolyticus*, 91.66% (44/48); *S. hominis*, 78.26% (36/46); *S. epidermidis*, 66.66% (40/60); *S. capitis*, 61.53% (8/13), *S. warneri*, 37.5% (3/8), *S. saprohyticus*, 50% (1/2), *S. lugdunensis*, 100% (2/2), and *S. sciuri*, 100% (1/1). The *mecA* gene was detected in 16 of 17 *sasX* gene-positive isolates (94.11%; p=0.056). The only *mecA*-negative and *sasX*-positive strain was a nasal colonizer* S. hominis* isolate.

### SCC*mec* types

With the protocol of Kondo et al. (17), 130 of the 135 *mecA*-positive isolates were typed. For all isolates, regardless of species, type V (34%) was the most prevalent, followed by type I (23.7%), type II (17%), type VI (10.4%), type III (4.4%), type I-II (1.5%), type I-III (1.5%), and type I-V (3.7%). Five of the isolates (3.7%) could not be typed. SCC*mec* distribution of the isolates is given in Table 6.

When all invasive and noninvasive isolates were taken into consideration, type II was more frequent than the other types in invasive isolates and found to be statistically significant (p=0.014). Also, the type II ratio was statistically significant (p=0.029) between invasive and noninvasive *S. epidermidis* isolates.

### Sequence analysis of *sasX* gene

Sequence analysis with *sasX* primers, *S. haemolyticus* isolates (n=9), and *S. capitis *isolate (n=1) gave high homology to proteins of* Staphylococcus* phage SPbeta-like, *S. epidermidis* RP62A, *S. aureus* strain XN108, *S. aureus subsp aureus* Z172, and *S. aureus subsp aureus* TW20 with the identities of 99%, 99%, 94%, 94%, and 94%, respectively. Sequence analysis of *S. hominis* isolates (n=3) was similar to that of *S. haemolyticus* and *S. capitis*, but the identities of the sequences changed to 94%, 94%, 99%, 99%, and 99%, respectively. The alignment descriptions of *S. epidermidis* isolates (n=4) were 94%, 99%, 99%, 99%, and 94%. Results of sequence analysis and related protein ID definitions with *sasX* primers are given in Table 7.

## DISCUSSION

*SasX*, a newly described protein, which is secreted by a gene containing a signal peptide and an LPXTG motif, was found at 127.2 kb ϕSPβ-like prophage of MRSA strain TW20. It is thought to play an important role in nasal colonization and MRSA virulence and is very similar to the *sesI* protein encoded by ϕSPβ prophage of *S. epidermidis* RP62A (19). De Backer et al. (20) stated geographical variations between Indian and European MRSA isolates harboring *sasX* and a newly described variant, *sasX-1*, from a European MRSA strain.

The potential role of *sasX* at colonization and virulence, distribution, and approaches about immunization is still being investigated by the researchers (10,21,22). Li et al. (10) found an increase of *sasX* among invasive MRSA isolates from 2003 to 2011, with low prevalence at community isolates, indicating that clonal spread is predominantly within the hospital setting.

A few data are available about the carriage of *sasX* in CoNS. Soumya et al. (23) investigated the *sasX* gene in 40 CoNS clinical isolates and found one positive *S. epidermidis* isolate obtained from blood. In their study, none of the *S. haemolyticus*, *S. hominis*, and *S. saprophyticus* isolates harbored the *sasX* gene. In our study, we found *sasX* carriage in *S. haemolyticus* (18.75%), *S. epidermidis* (6.66%), *S. hominis* (6.52%), and *S. capitis* (7.69%) isolates. De Backer et al. (20) investigated a total of 32 CoNS; *S. epidermidis* (n=22), *S. capitis* (n=4), and *S. hominis* (n=6) were isolated from endotracheal tubes, but *sasX* could not be found (20). The different rates of *sasX* carriage in different species can be explained by the distribution of the isolates in different geographies and a variety of clinical samples.

The *sasX* gene was thought to facilitate the nasal colonization of MRSA (10). In our study, we found *sasX* carriage of CoNS at a rate of 7.69% of the isolates (*n*=26) from nasal vestibules of healthy volunteer hospital workers. It is well known that healthcare professionals have more frequent nasal colonization of *S. aureus* and other resistant CoNS, and because the *sasX* gene may facilitate the nasal colonization, the possible role of this gene in healthcare-related settings for nasal colonization should be investigated.

When our findings are considered in respect of invasive and noninvasive isolates, the carriage of *sasX* is found in 12.36% of isolates from peripheral blood cultures, and 11.43%, 7.69%, and 0% of isolates from central venous catheter (catheter colonization), nasal vestibule, and hands, respectively. Our peripheral blood and nasal isolate results were in concordance with the results of Li et al. (10). The high *sasX* ratio in catheter colonizers seems to support the role of *sasX* in adherence to surfaces, as in nasal isolates. The small number of CoNS rather than *S. epidermidis*, *S. haemolyticus*, and *S. hominis* isolates and the absence of tracheal aspirate and abscess samples were the limitations of our study.

Also, Li et al. (21) stated that invasive infections caused by *sasX*-positive isolates were higher than *sasX*-negative isolates in accordance with our isolates from blood cultures. There was no *sasX* carriage in isolates from hands, and this finding also supports the results of Li et al. (10,20), which underlined the potential role of *sasX* in invasive rather than noninvasive isolates.

Sequence analysis of the *sasX* gene of the isolates showed 99% homology with related proteins of *Staphylococcus* phage SPbeta-like and *S. epidermidis* RP62A, especially for *S. haemolyticus* and *S. capitis* isolates. *S. hominis* and *S. epidermidis* isolates had 94% and 99% homology to related proteins of *Staphylococcus* phage SPbeta-like and *S. epidermidis* RP62A, respectively, indicating that the phages of CoNS rather than *S. epidermidis* isolates were also highly conserved.

In a study about MRSA ST239 isolates, Holden et al. (19) stated that the *sasX* gene is located at the 3′ end region of a ϕSPβ-like prophage, which had coding sequences for aminoglycoside resistance. In our study, no difference was found for gentamycin and other antimicrobial resistance between *sasX-*positive and *-*negative isolates in accordance with Li et al. (21).

In our study, type V SCC*mec* (34%) was the most prevalent type in all isolates regardless of species, except *S. hominis* (Table 6). The SCC*mec* types differ among CoNS species and between studies. McManus et al. (24) reported that type IVc is the most common type in methicillin-resistant *S. epidermidis* isolated from cerebrospinal fluid and external ventricular drains in device-associated meningitis patients. Garza-González et al. (25) found that types III and IVa were the predominant types in *S. epidermidis* isolated from blood samples, whereas Saffari et al. (26) and Chen et al. (27) found type IV and type III/SCCHg, respectively. Pinheiro et al. (28) characterized *S. epidermidis* (n*=*79) blood culture isolates and found SCC*mec* types III (53.2%) and II (29.1%) as the most prevalent types. The diversity of the types found in these studies may depend on the geographical differences, and the clinical samples from which the isolates were obtained, the epidemiologic similarity of the isolates, and the methodological limitations were used to detect SCC*mec *types in CoNS. In our study, no statistical difference was found between the presence of the *sasX* gene and the SCC*mec *type.

In our study, with respect to invasive isolates, SCC*mec* type II was more frequent than the other types when compared with noninvasive isolates, and it was found to be statistically important (p=0.014). Also, the type II ratio was found to be statistically important (p=0.029) between invasive and noninvasive *S. epidermidis* isolates. In some studies, it is seen that there is no significant difference in invasive and noninvasive isolates in terms of SCC*mec* type (9,29). It is thought that this difference may be due to the number of isolates and diversity of sample origin (cat, pig, human isolates, etc.) used in the studies.

Some CoNS isolates could not be typed in our study with the multiplex PCR method of Kondo et al. (17). As Garza-González et al. (30) also stated in their study, new methodologies are needed to be designed because of the inconsistent results for SCC*mec* typing of CoNS strains. The usage of two or more different multiplex PCR protocols/conditions (new primer designs for conserved gene sequences) directed to different targets of the SCC*mec* complex may be helpful to solve this issue.

In this study, we characterized our invasive and noninvasive CoNS isolates from different samples of human origin and for the presence of *sasX* gene and SCC*mec* types. For *sasX*, we found 9.44% positivity. Remarkably, the *sasX* gene was found to be negative from strains isolated from healthy volunteers’ hands, which indicate the possible role of this gene in invasive isolates. No statistical difference was found between *sasX*-positive and -negative isolates in respect to antibacterial resistance and between* sasX* presence and SCC*mec *types. SCC*mec* type V is the most prevalent type of *S. haemolyticus (*47.7%) and *S. epidermidis *(35%) isolates. As far as we know, this is the first data about the distribution SCC*mec* types and *sasX* carriage of different CoNS from our country.

As a result, more large scale and comprehensive studies are needed to define the SCC*mec* type distribution and to find out the molecular epidemiological characteristics of *sasX* and its possible role at colonization and infection in particular species. Also, more clinical samples of CoNS are needed.

## Figures and Tables

**Table 1:**
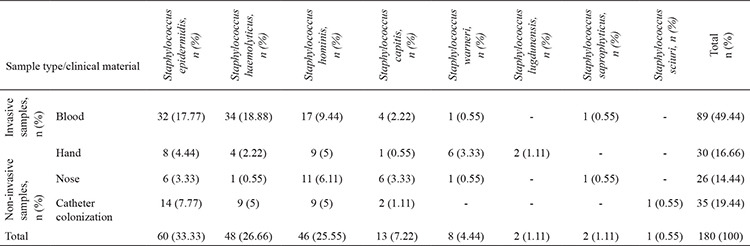
Distribution of the strains according to the samples

**Table 2:**
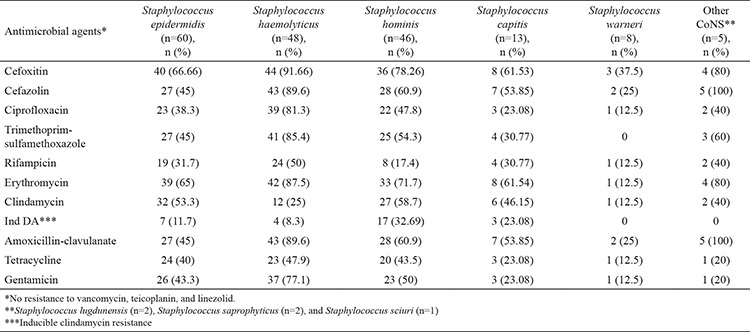
Resistance patterns of coagulase-negative staphylococci

**Table 3:**
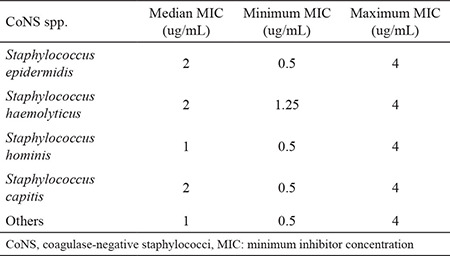
Minimum inhibitor concentration values of vancomycin

**Table 4:**
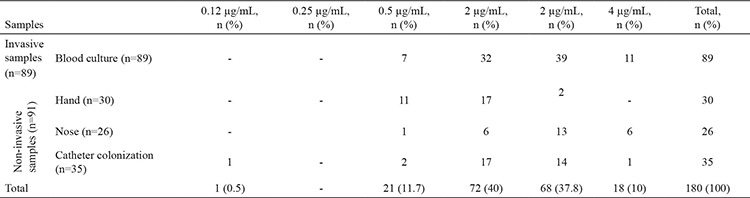
Minimum inhibitor concentration ranges of vancomycin

**Table 5:**
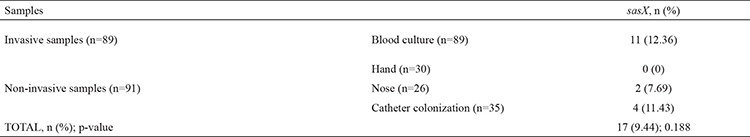
Distribution of *sasX* carriage rates and p-value according to the sample types

**Table 6:**
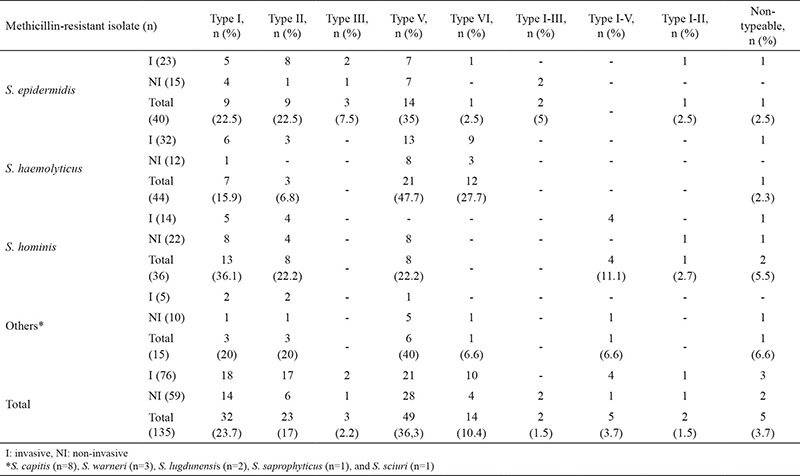
SCC*mec* distribution of the isolates

**Table 7:**
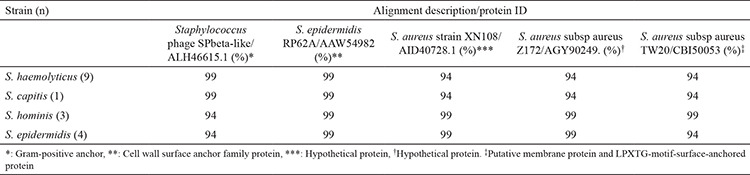
Alignment description and related protein ID definition results of *sasX* gene sequencing
